# Intraoral onlay block bone grafts versus cortical tenting technique on alveolar ridge augmentations: a systematic review

**DOI:** 10.4317/medoral.25169

**Published:** 2022-02-20

**Authors:** Amparo Aloy-Prósper, Esther Carramolino-Cuéllar, David Peñarrocha-Oltra, David Soto-Peñaloza, Miguel Peñarrocha-Diago

**Affiliations:** 1 Assistant Professor of Oral Surgery, Stomatology Department, Faculty of Medicine and Dentistry, University of Valencia, Valencia, Spain. Professor Faculty of Dentistry, European University of Valencia, Valencia, Spain; 2 Doctor of Dentistry, Stomatology Department, Faculty of Medicine and Dentistry, University of Valencia, Valencia, Spain. Professor Faculty of Dentistry, European University of Valencia, Valencia, Spain; 3 Titular Professor of Oral Surgery, Stomatology Department, Faculty of Medicine and Dentistry, University of Valencia, Valencia, Spain; 4 Doctor of Dentistry, Stomatology Department, Faculty of Medicine and Dentistry, University of Valencia, Valencia, Spain; 5 Chairman of Oral Surgery, Stomatology Department, Faculty of Medicine and Dentistry, University of Valencia, Valencia, Spain

## Abstract

**Background:**

To review systematically the bone gain and superficial resorption rate of the onlay block bone grafts versus the cortical tenting technique, as well as secondarily study the postoperative complications, implant survival and success rates, and peri-implant marginal bone loss.

**Material and Methods:**

Following the recommended methods for systematic reviews and meta-analyses (PRISMA), an electronic search was performed in the PubMed (MEDLINE), EMBASE and the Cochrane Library of the Cochrane Collaboration (CENTRAL) databases to identify all relevant articles published up to March 2021 on onlay block bone grafts and cortical tenting technique.

**Results:**

Eighteen papers complied with the inclusion criteria. In onlay grafts, the vertical bone gain mean was 4.24 mm, and resorption 20.91%; and 4.29 mm in the horizontal augmentation with a resorption of 10.28%. The complication rate was 14.8%. The implant survival and success rates were 100% and 92%; and the mean peri-implant bone loss ranged from 0.6 to 1.26 mm.

In cortical tenting technique, the vertical bone gain mean was 6.17 mm and the resorption of 9.99%; and 5.55 mm in the horizontal augmentation with a 6.12% of resorption. The complication rate was 24.6%. The implant survival and success rates were 96.63% and 100%; and the mean peri-implant bone loss ranged from 0.27 to 0.77mm.

**Conclusions:**

Despite the limitations, both techniques offer a predicTable way to reconstruct atrophic alveolar ridges, though the cortical tenting technique seems to achieve a greater bone gain and a lower surface resorption. Current evidence is still limited due to the inadequate follow-up, lack of information referred to methodological quality and sample attrition.

** Key words:**Onlay graft, cortical graft, intraoral bone, augmentation procedure.

## Introduction

Onlay block bone grafts permit to correct anomalous intermaxillary relationships and achieve an adequate bone in terms of volume and morphology allowing the fixation of the implants in an aesthetic and functional way (1). Autogenous bone graft has been described as the best bone substitute due to its biological properties (2,3); in particular, harvesting intraoral donor areas for the treatment of alveolar defects of the partially edentulous patients has been justified by presenting less severe and more localized defects (4).

However, an important problem of alveolar reconstruction with appositional block grafts (onlay grafts) is its high incidence of superficial resorption in the medium and long term (5,6), which could lead to a lower bone gain and diminish the augmentation procedure results (7). This resorption is particularly important in vertical augmentation as the forces exerted on the graft, when the soft tissue sheath expands vertically, may cause even greater surface resorption (8). The vertical augmentation represents the most challenging procedure for clinicians from a biological point of view, due to a lower source of osteogenic cells, less vascularization, greater difficulty in achieving primary closure, and an important risk of wound dehiscence (9). A bone loss in height of 41.5% of the volume obtained in the first 6 months has been reported with onlay grafts (10). Pikos *et al*. (11) reported a bone resorption after augmentation with onlay bone blocks greater than 40% of the volume, and Cordaro *et al*. (7), found a graft resorption rate after a healing period of 5 to 6 months from 23.5% to 42% (23.5% in width and 42% in height). The process of graft neovascularization that occurs after fixation in the recipient area is essential for its long-term viability; this revascularization, which must be fast and complete, is difficult to occur in its entirety in the case of bone blocks with a large cortical component such as onlay grafts (9). It has been described that before neoformed vessels reach the interior of the graft, necrosis phenomena of the central areas had already occurred, evidenced by the arrival of macrophage cells, which eventually resulted in their subsequent resorption. The cortical tenting technique consists of a thin lamina cortical block tented over the particulate material (12,13). In this technique, the graft structure combines an outer layer of thin cortical bone, acting as an "autogenous biological membrane", which provides consistency while allowing vascular penetration into the interior of the particulate bone, which could favor rapid revascularization and reduce the resorption (14,15). In addition, by screwing the bone block at a certain distance from the alveolar crest may lead to high bone augmentation, unlike the onlay graft in which the increase is limited by the width of the block harvested (16). The study was based on the hypothesis that the cortical tenting technique will achieve greater bone gain and less superficial graft resorption compared to onlay grafts in patients with bone atrophy of the alveolar ridge.

 The aim of this study was to systematically review the following question: In patients with localized alveolar ridge defects who underwent a bone augmentation procedure with intraoral autogenous block bone grafts, the cortical tenting technique achieves better outcomes in terms of bone gain and superficial resorption rate than onlay block grafts? This was done by firstly assessing the bone gain and resorption rate measurements, and secondly evaluating complications related to the augmentation procedure, implant survival, implant success, and radiographic peri-implant marginal bone loss.

## Material and Methods

This systematic review complies with the PRISMA statement (Preferred Reporting Items for Systematic reviews and Meta-Analyses) (11). The review protocol was registered and allocated the identification number CRD42020187085 in the PROSPERO International Prospective Register of Systematic Reviews hosted by the Centre for Reviews and Dissemination, University of York, National Institute for Health Research (United Kingdom).

- Focus question

The focus question was established according to an adaptation of the PICO structured question, in this case applying a PEO (population, exposition, outcome) format, and considering the importance of including observational studies without a comparative group, such as single cohort studies. This approach is adequate for performing qualitative systematic reviews in health interventions.

P (population): Edentulous patients with atrophic alveolar ridges.

E (exposition): Intraoral autogenous block bone grafts: onlay grafts and/or cortical tenting technique augmentation.

O (outcome):

O1: Bone gain and resorption rate.

O2: Postoperative complications related to the bone graft augmentation: neurosensory disturbances, membrane or screw exposures, wound dehiscence, infection, severe bone resorption.

O3: Survival and success implant rates, and peri-implant marginal bone loss.

- Information sources and data search

An automatized electronic and manual literature searches were conducted in three major electronic databases: MEDLINE (via PubMed), EMBASE, and the Cochrane Library of the Cochrane Collaboration (CENTRAL) with the following keywords: ´partial edentulous patients´, ´edentulous patient´, ´edentulous jaw´, ´edentulous maxilla´, ´edentulous mandible´, ´atrophied jaw´, ´jaw atrophy´, ´bone graft´, ´bone regeneration´, ´bone augmentation´, ´vertical ridge augmentation´, ´splitter bone graft technique´, ´khoury technique´, ´bilaminar technique´, ´shell technique´, ´autogenous bone´, ´autologous bone´, ´intraoral bone´, ´intraoral onlay block´, ´onlay bone graft´, ´block graft´, ´bone block graft´, ´bone gain´, ´resorption´. The search contemplated papers published without language restrictions up to March 2021. The search strategy included a combination of the controlled terms (MeSH and EMTREE), and keywords were used whenever possible in an attempt to obtain the best search results. The following search strategy in Pubmed was carried out: (("Jaw, Edentulous"[Mesh] OR ( "Jaw, Edentulous, Partially"[Mesh] OR "Mouth, Edentulous"[Mesh] OR partial edentulous patients OR edentulous patient OR edentulous jaw OR edentulous maxilla OR atrophied jaw OR jaw atrophy) AND (bone graft OR bone regeneration OR bone augmentation OR vertical ridge augmentation OR VRA OR lateral ridge augmentation OR HRA) AND ((splitter bone graft technique OR khoury technique OR bilaminar technique OR shell technique) OR ((autogenous bone OR autologous bone OR intraoral bone) AND (intraoral onlay block OR onlay bone graft OR block graft OR bone block graft))) AND (bone gain OR resorption *) NOT sinus).

The following search strategy in Embase was carried out: (((('edentulousness'/exp OR 'edentulousness' OR edentulous) AND ('jaw'/exp OR jaw) OR atrophied) AND ('jaw'/exp OR jaw) OR 'jaw'/exp OR jaw) AND ('atrophy'/exp OR atrophy) OR 'maxilla atrophy'/exp OR 'maxilla atrophy' OR 'bone atrophy'/exp OR 'bone atrophy' OR 'mandible atrophy'/exp OR 'mandible atrophy') AND ((((splitter AND ('bone'/exp OR bone) AND ('graft'/exp OR graft) AND ('technique'/exp OR technique) OR khoury) AND ('technique'/exp OR technique) OR bilaminar) AND ('technique'/exp OR technique) OR 'shell'/exp OR shell) AND ('technique'/exp OR technique) OR (((('onlay graft'/exp OR 'onlay graft' OR block) AND ('graft'/exp OR graft) OR 'bone'/exp OR bone) AND block AND ('graft'/exp OR graft) OR intraoral) AND onlay AND block)) AND (('bone'/exp OR bone) AND gain OR 'resorption'/exp OR resorption).

As a complement, a manual search of main primary source related topic was performed including: Journal of Clinical Periodontology, Clinical Oral Implants Research, Clinical Implant Dentistry and Related Research, Clinical Oral Investigations, Journal of Periodontology, International Journal of Oral & Maxillofacial Implants, Journal of Oral and Maxillofacial Surgery. Finally, the reference lists of preselected articles were checked to find possible eligible studies not detected through electronic sources.

- Eligibility criteria

Articles were included in this systematic review if they met the following inclusion criteria.

- Study design: randomized clinical trials, prospective and retrospective cohort studies, studies on humans, ≥5 patients; publication in English or Spanish, up to March 2021.

Patient: patients with atrophic alveolar ridges treated with intraoral autogenous block bone grafts.

Intervention: onlay block bone grafts or cortical tenting technique, with a minimum follow-up of 6 months after augmentation procedure.

Outcomes: Studies that include data related to the bone gain and/or resorption rate as main variables. And as secondary variables: postoperative complications related to the augmentation procedure, survival and success rates and peri-implant marginal bone loss.

Reviews, case reports, letters or comments to the editor, expert reports, or studies on sinus augmentation or other regenerative procedures not specified in inclusion criteria were excluded. No restrictions were placed on the year of publication. Authors were contacted for clarification of missing information when necessary. If a study included both intraoral/extraoral autologous bone grafts, only the data of intraoral blocks was considered.

- Search strategy

**Figure 1:**
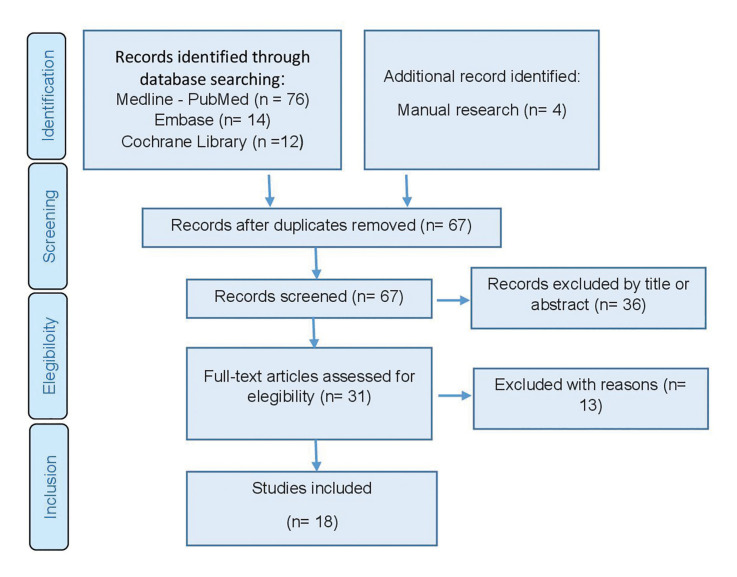
PRISMA flowchart of searching and selection process of titles during systematic review.

The search strategy was carried out by two independent reviewers (AAP, ECC). Publications that did not meet the inclusion criteria were excluded. In the case of disagreement, consensus was reached through discussion with a third reviewer. In the first phase, titles were screened in order to eliminate irrelevant publications. In the second phase, abstracts were filtered according to the number of patients, the type of graft, the intervention and the outcome characteristics. The studies without enough information or with unstructured abstracts to determine its exclusion were deemed for full-text assessment. The third phase consisted of a full reading of each text using a predetermined data extraction form to confirm study eligibility upon the predetermined inclusion and exclusion criteria (Fig. 1).

The level of agreement regarding inclusion of potential studies was calculated by k-statistics for the second and third phase of screening.

- Extraction data

Evidence Tables were created with the study data. The following data were collected from the publications: type of study, type of procedure, number of patients, number of implants, donor site of the grafts, number of grafts, grafting site, initial bone defect (mm), intraoperative augmentation (mm), postoperative augmentation (mm) total bone gain (mm), resorption rate (% and mm), follow-up (months), number and type of postoperative complications above described, implant survival and success rates and mean marginal bone loss (mm).

- Quality and risk of bias assessment

Two reviewers independently and in duplicate evaluated the quality of the included studies. The methodological quality of observational studies was assessed with the Newcastle-Ottawa Scale (14), and the Cochrane Collaboration tool for assessing the risk of bias was employed for the assessment of randomized controlled trials (RCTs). For each aspect of the quality assessment, the risk of bias was scored following the recommendations of the Cochrane Handbook for Systematic Reviews of Interventions 5.1.0 (http://handbook.cochrane.org). The judgment for each entry consisted of recording “yes” (low risk of bias), “no” (high risk of bias) or “unclear” (either lack of information or uncertainty over the potential for bias). The publications were grouped into the following categories: (A) low risk of bias (possible bias not seriously affecting the results) if all the criteria were met; (B) high risk of bias (possible bias, seriously weakening the reliability of the results) if one or more criteria were not met. The level of agreement was calculated by k-statistics for the second and third stage of screening.

- Data synthesis

With the aim of summarizing and comparing studies, mean data on main variables were grouped for each study group. As the mean data found in the analyzed studies came from samples with different grafting sites and number of implants, weighted arithmetic mean was calculated to obtain feasible outcomes. A meta-analysis was not able to be perfomed due to the lack of randomized studies comparing both procedures.

## Results

- Study selection

A total of 102 articles were obtained from the initial screening process: Medline - PubMed (n=76), EMBASE (n=14) and the Cochrane Library (n=12). In addition, 4 titles were obtained through manual searching (references list and primary sources). Of these publications, 31 were identified as potentially eligible articles through screening by titles and abstracts. The full-text articles were subsequently obtained and thoroughly evaluated. As a result, 18 articles fulfilled the inclusion criteria and were finally included in the present systematic review (Fig. 1). The k value for inter-reviewer agreement for study inclusion was 0.87 (titles and abstracts) and 1.0 (full texts) indicating ‘‘good’’ and ‘‘complete’’ agreement, respectively.

Study characteristics

Finally 4 randomized clinical trial were included (RCT) (13,17-19), 6 prospective studies (9,15,20-23), 1 prospective case series (8), 3 retrospective studies (24-26) and 4 retrospective case series (7,12,27,28). Patient was the unit at random.

A total of 470 patients and 581 sites were treated (cortical tenting: 237 patients, 256 sites; onlay block grafts: 233 patients, 325 sites) (Table 1).

- Risk of bias

**Figure 2:**
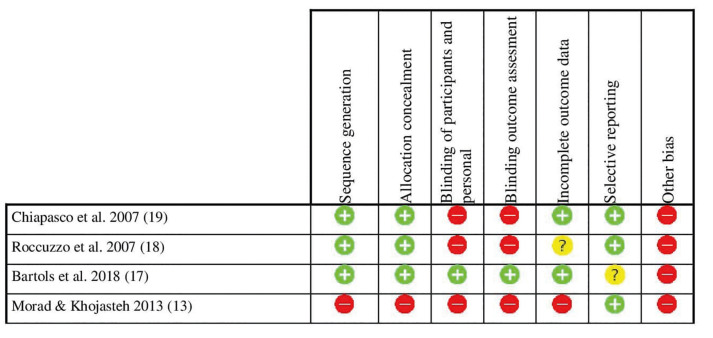
Randomized studies risk of bias following Cochrane´s guidelines.

**Figure 3:**
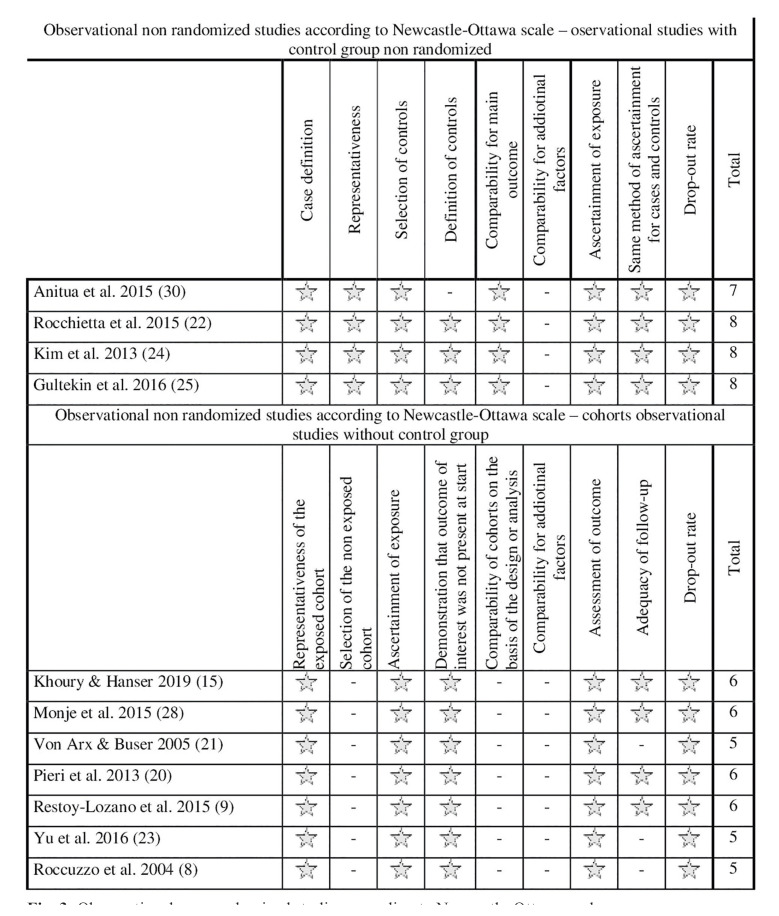
Observational non-randomized studies according to Newcastle-Ottawa scale.

For randomized studies, a high risk of bias was considered (Fig. 2). For non-randomized observational studies, the risk of bias was considered low in 4 studies and high in 7 studies (Fig. 3). Substantial inter-rater agreement was obtained according to the Cohen kappa test, k = 0.78 (95% confidence interval), based on the Landis & Koch scale (29).

**Table 1:**
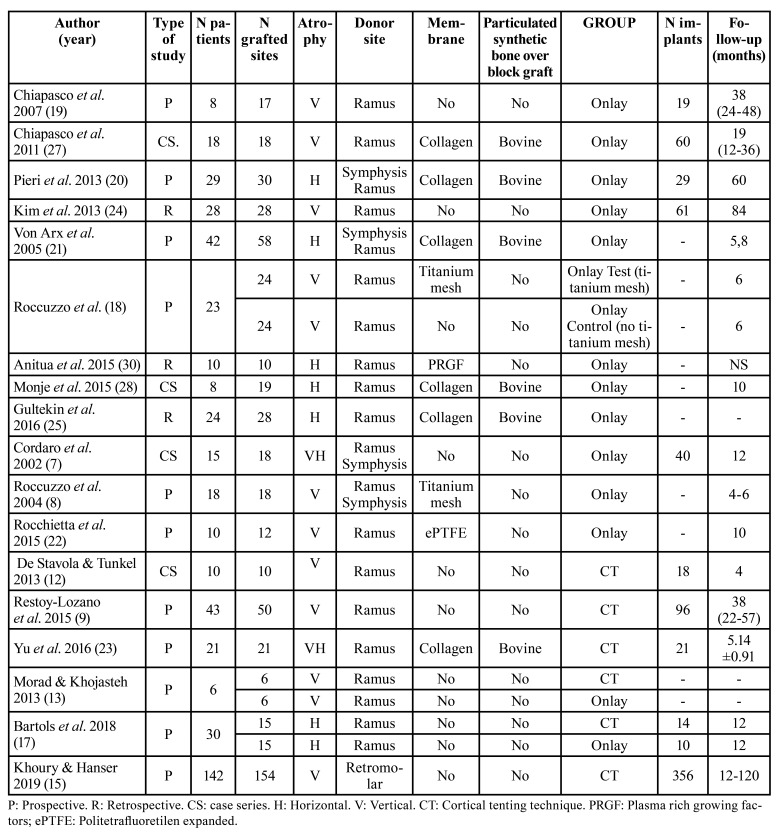
Characteristics of the included studies.

- Synthesis of results

Bone gain and superficial bone resorption

In relation to onlay block bone grafts, 14 studies provided data on bone gain (7,8,13,17-22,24,25,27,28,30). Vertical bone gain mean was 4.24 mm, ranged from 2.2 (SD 0.66) (7) to 6.5 mm (SD 2.3) (24). Superficial resorption rate mean was 20.91%, ranged from 6.14% to 42%. Studies that none membrane was used showed the lowest bone gain values [2.2 (7) to 4mm (19)], and the higher values of resorption (34.5% (18) to 42% (7)). Horizontal bone gain mean was 4.29 mm, ranged from 3.1mm (SD 1.75) (30) to 5.1mm (SD 1.6) (17). Superficial resorption rate mean was 10.28%, ranged from 7.2% (21) to 23.5% (7). The highest value was reported from Cordaro *et al*. (7) on 18 grafted sites in which none membrane was used.

Regarding the cortical tenting technique, 6 studies provided data on bone gain (9,12,13,15,17,23). Vertical bone gain mean was 6.17mm, ranged from 5.12mm (SD 1,05) (23) to 7.3mm (SD 2.6) (15). Superficial resorption rate mean was 9.99%, ranged from 4.6% (12) to 18.3% (13). The higher values were reported from De Stavola y Tunkel (12) and Restoy-Lozano *et al*. (9) who used a tunnel approach and made a shell with the blocks fixed on occlusal an buccal. Horizontal bone gain mean was 5.55mm, ranged from 4.63 (SD 1.5) (17) to 7.93 (SD 0.92) (23). Superficial resorption rate mean was 6.12%, ranged from 6.15% (23) to 22% (17). Descriptive outcomes on bone gain and superficial resorption are shown in Table 2.

**Table 2:**
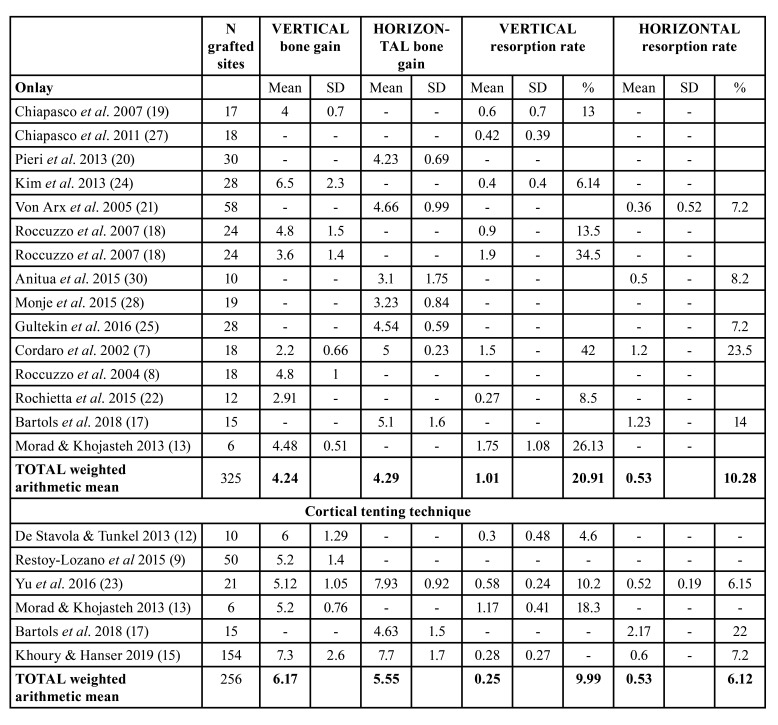
Descriptive outcomes on bone gain and superficial resorption.

Complications related to bone grafting

Ten studies reported data on complications related to onlay block bone grafting procedure (7,8,13,17-20,24,25,27) and 6 to the cortical tenting technique (9,12,13,15,17,23). Thirty complications on 202 grafting sites were reported to onlay grafts versus 63 complications on 193 grafting sites to cortical tenting technique (14.8% versus 24.6%, respectively) (Table 3). In onlay group, the most frequent complication of the recipient area was wound dehiscence with early membrane exposure (18,20) and/or graft exposure (19,20). In cortical tenting technique, the most frequent complication was wound dehiscence (9,15,17,23), following by screw exposures (15), graft loss (9,17), and infection (9). Chin hypoesthesia was the most frequent complication of the donor area in both groups.

Implant survival and success rates ana peri-implant marginal bone loss

To onlay group, implant survival and success rates means were 100% and 92%, respectively after 48 months post-loading [12-84] and peri-implant marginal bone loss mean was 0.6 mm at 12 months post-loading and 1.26 mm at 7-years post-loading. To cortical tenting technique group, implant survival and success rates means were 96.63% and 100%, respectively after 32 months post-loading [12-86] and peri-implant marginal bone loss mean was 0.27 mm at 12 months post-loading and 0.77 mm at 48-84 months post-loading (Table 4).

**Table 3:**
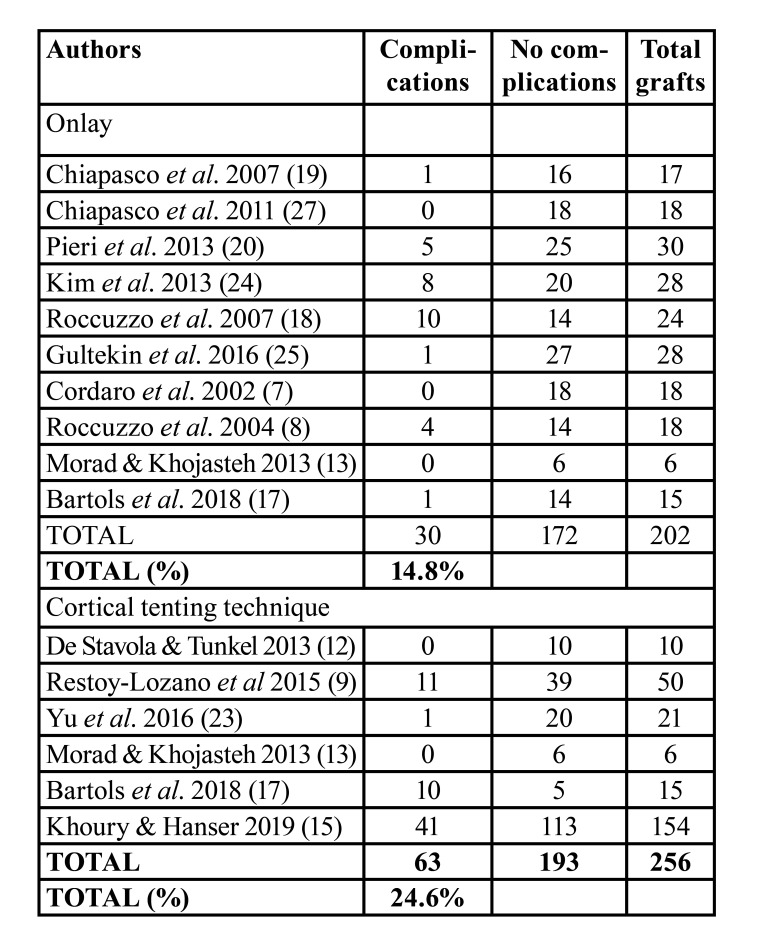
Descriptive outcomes on postoperative complications related to bone grafting procedure.

**Table 4:**
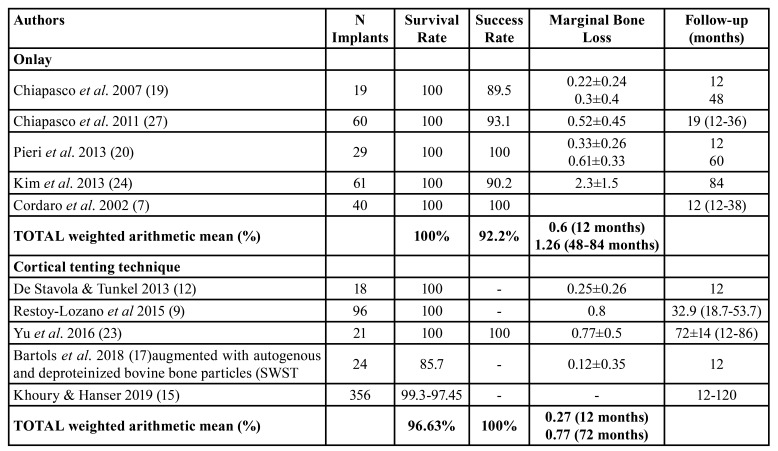
Descriptive outcomes of implant survival and success rate and marginal bone loss.

## Discussion

- Main findings

The present systematic review elucidates the bone gain, superficial resorption and postoperative complications rate, and implant outcomes (survival and success rates and peri-implant marginal bone loss) from onlay block grafts versus cortical tenting technique.

The lack of randomized studies comparing both techniques made impossible perform a meta-analysis so the outcomes were shown in a descriptive way for each group.

- Bone gain and resorption rate

The main disadvantage of bone reconstruction procedures, mainly in the vertical augmentation, is the resorption of a significant proportion of the graft (31). Several clinical studies have reported a graft resorption rate from 11-34% at 4 to 6 months after surgery (18,19,32) up to 42% (7). This percentage of resorption represents a substantial loss of bone gain. According to Morad & Khohasteh (13) the cortical block lamina screwed into occlusal would act as a protection of the particulate graft by releasing it from the tensions that soft tissue exerts. According to De Stavola and Tunkel (12) the particulate graft filling of the gap between the thin cortical bone (screwed at a certain distance) and the crest would improve the revascularization of the area, which could explain the lower resorption and the greater bone gain. Stavola and Tunkel (12) and Restoy-Lozano *et al*. (9) reported the greatest bone gain and the lowest resorption rate when performing the technique with a tunnel approach, a factor that could be even more influenced towards a better result, because of the tissue integrity which may provide a greater supply of blood for regeneration. Mazzocco *et al*. (33) did not provide data regarding bone resorption, but they agreed that maintaining the integrity of the periosteum should have a positive effect on maintaining volume. Similarly, the results of the present review also showed higher values ​​of bone gain in cortical tenting technique group (all studies over 5mm), although it is worth mentioning that both techniques achieved good bone gain results.

- Postoperative complications related to the bone grafting procedure

The vertical augmentation techniques have been associated with high complication rates, ranging from 8% to 90% (34). Urban *et al*. (35) reported a complication rate of 23.9% for vertical bone block grafting procedures. In the present systematic review, complication rates were of 14.8% for onlay grafts and 24.6% for cortical tenting. The wound dehiscence (36) and the transient paraesthesia of the mental nerve (34) have been reported the most commonly complication in the studies of autogenous block grafting. Similarly, in the present study the most frequent complication of the recipient area was the dehiscence of the wound with or without exposure of the graft; and the most frequent complications of the donor zone was chin paresthesia, mainly related grafts harvested from symphysis. Both techniques therefore presented similar complications. However, it should be mentioned that the cortical tenting technique would need a greater learning curve due to the added difficulty of handling the graft block which must be cut into two thin sheets and be frailer to manipulate.

- Implant outcomes 

Different systematic reviews comparing various vertical bone augmentation procedures found no evidence of any procedure having a greater benefit with respect to implant failure (34,36). Camps-Font *et al*. (34) reported a success/survival of implants with block grafting techniques around 90%. In the present study high implant survival rates were reported in both groups (onlay: 92.2%-100%; cortical tenting: 96.63-100%). Implant success rate was poorly referenced in the studies reviewed; this could be due to the lack of uniform criteria for considering the definition of implant success. The lower peri-implant marginal bone loss in cortical tenting technique could be related to the better vascularization of the particulate graft that could imply a greater stability of long-term hard tissues. However, the lack of randomized controlled clinical studies comparing both techniques makes it difficult to obtain definitive conclusions. Despite the limitations, both techniques offer a predicTable way to reconstruct atrophic alveolar ridges, though the cortical tenting technique seems to achieve a greater bone gain and a lower surface resorption. However, current evidence is limited due to the inadequate follow-up, lack of information referred to methodological quality and sample attrition.
